# Direct observation of respectful maternity care in five countries: a cross-sectional study of health facilities in East and Southern Africa

**DOI:** 10.1186/s12884-015-0728-4

**Published:** 2015-11-23

**Authors:** Heather E. Rosen, Pamela F. Lynam, Catherine Carr, Veronica Reis, Jim Ricca, Eva S. Bazant, Linda A. Bartlett, on behalf of the Quality of Maternal and Newborn Care Study Group of the Maternal and Child Health Integrated Program

**Affiliations:** Johns Hopkins Bloomberg School of Public Health, Baltimore, MD USA; Jhpiego/Kenya, Nairobi, Kenya; Jhpiego/Baltimore, Baltimore, MD USA; Jhpiego/Mozambique, Maputo, Mozambique

**Keywords:** Respectful maternity care, Quality of care, Disrespect, Maternal health, Ethiopia, Kenya, Madagascar, Rwanda, Tanzania, Zanzibar

## Abstract

**Background:**

Poor quality of care at health facilities is a barrier to pregnant women and their families accessing skilled care. Increasing evidence from low resource countries suggests care women receive during labor and childbirth is sometimes rude, disrespectful, abusive, and not responsive to their needs. However, little is known about how frequently women experience these behaviors. This study is one of the first to report prevalence of respectful maternity care and disrespectful and abusive behavior at facilities in multiple low resource countries.

**Methods:**

Structured, standardized clinical observation checklists were used to directly observe quality of care at facilities in five countries: Ethiopia, Kenya, Madagascar, Rwanda, and the United Republic of Tanzania. Respectful care was represented by 10 items describing actions the provider should take to ensure the client was informed and able to make choices about her care, and that her dignity and privacy were respected. For each country, percentage of women receiving these practices and delivery room privacy conditions were calculated. Clinical observers’ open-ended comments were also analyzed to identify examples of disrespect and abuse.

**Results:**

A total of 2164 labor and delivery observations were conducted at hospitals and health centers. Encouragingly, women overall were treated with dignity and in a supportive manner by providers, but many women experienced poor interactions with providers and were not well-informed about their care. Both physical and verbal abuse of women were observed during the study. The most frequently mentioned form of disrespect and abuse in the open-ended comments was abandonment and neglect.

**Conclusions:**

Efforts to increase use of facility-based maternity care in low income countries are unlikely to achieve desired gains if there is no improvement in quality of care provided, especially elements of respectful care. This analysis identified insufficient communication and information sharing by providers as well as delays in care and abandonment of laboring women as deficiencies in respectful care. Failure to adopt a patient-centered approach and a lack of health system resources are contributing structural factors. Further research is needed to understand these barriers and develop effective interventions to promote respectful care in this context.

## Background

Increasing access of pregnant women to skilled care during childbirth is a key strategy for reducing maternal and early neonatal mortality and morbidity. Most maternal deaths are considered preventable [1] and the majority could be averted by increased access to a skilled care provider supported by the resources of a functioning health system [2]. Recent modeling of the effect of scaling up selected evidence-based interventions during facility-based labor and delivery confirms a 79 % decrease in maternal deaths is possible [3]. With the global agenda historically focused on increasing access, or quantity, of skilled care, the need to improve quality of care has received less attention [4, 5]. To save women’s lives and improve maternal and newborn health, women must both come to the facility to give birth with a skilled health provider and receive high quality care to prevent and address complications that may arise.

Quality of care encompasses structure, processes of care, and outcomes [6]. Structural elements include the presence of needed medicines, equipment, and provider training while outcomes are changes in health status and patient satisfaction. Processes of care include both technical aspects, which is the delivery of clinical procedures and treatments, and the client-provider interpersonal relationship including how information is shared and decisions about care are made [7]. The personal interaction between client and provider is important in shaping women’s experiences and their perceptions of maternity care [8]. Poor interpersonal communication between client and provider during maternity care at health facilities in low resource settings is increasingly recognized as a barrier to accessing skilled care for routine and complicated births [9, 10]. Women and their families especially mention rude and uncaring provider attitudes, lack of privacy, discrimination against cultural practices, physical abuse, dirty facilities, and delays in receiving care as reasons for dissatisfaction with facility services or for not giving birth at facilities nor seeking facility-based care for complications [11–16].

An increasingly cited framework for describing interpersonal aspects of care during childbirth is the seven domains of disrespect and abuse (D&A) outlined in Bowser and Hill’s landscape evidence review: physical abuse; non-consented care; non-confidential care; non-dignified care; discrimination; abandonment of care; and detention in facilities [17]. The White Ribbon Alliance subsequently published the Respectful Maternity Care Charter: The Universal Rights of Childbearing Women, grounded in international human rights instruments such as the Universal Declaration of Human Rights [18]. The seven articles of the Charter are closely aligned to the seven domains of D&A (see 'Seven rights') [19]. While these approaches are similar, the Charter frames the discussion in terms of positive, desired behaviors. The concept of respectful maternity care (RMC) acknowledges that women’s experiences of childbirth are vital components of health care quality and that their “autonomy, dignity, feelings, choices, and preferences must be respected [19].” RMC has commonalities with other efforts to refocus medical care away from a disease-oriented model which privileges the physician as expert including patient-centered care and the humanization of childbirth [20, 21].

- Seven rights of childbearing women from Respectful Maternity Care Charter [18].
- Article 1. Every woman has the right to be free from harm and ill treatment.
- Article 2. Every woman has the right to information, informed consent and refusal, and respect for her choices and preferences, including companionship during maternity care.
- Article 3. Every woman has the right to privacy and confidentiality.
- Article 4. Every woman has the right to be treated with dignity and respect.
- Article 5. Every woman has the right to equality, freedom from discrimination, and equitable care.
- Article 6. Every woman has the right to healthcare and to the highest attainable level of health.
- Article 7. Every woman has the right to liberty, autonomy, self-determination, and freedom from coercion.

There is limited evidence on the prevalence of respectful care or D&A in facility-based maternity services delivered in low-resource settings [17, 22]. Neither routine health information systems nor facility assessments such as the Service Provision Assessment (SPA) capture this type of data [23]. Four recent studies in Kenya, Tanzania, Ethiopia, and Nigeria analyzed women’s experiences during childbirth to estimate prevalence of disrespect and abuse (20 %, 20–28 %, 78, and 98 %, respectively) [24–27]. Our team conducted a study of quality of care at health facilities in five countries in East and Southern Africa with a focus on clinical procedures for prevention, identification, and management of the most common causes of maternal and newborn mortality during childbirth. Although the study was not designed with a specific plan to assess respectful care or D&A during labor and delivery, patient-centered care was one of the dimensions of quality evaluated. To meet the research gap, we applied the lens of women’s rights and the Respectful Maternity Care Charter to relevant data in the quality of care study. The goal of this paper is to provide a descriptive overview of the quality of respectful maternity care in diverse facility settings in East and Southern Africa.

## Methods

### Study design, context, and sampling

This is an analysis of select data from a series of cross-sectional surveys implemented in 2009–2012 by the Maternal and Child Health Integrated Program (MCHIP) to assess quality of care in Ethiopia, Kenya, Madagascar, Rwanda, and the United Republic of Tanzania. In each country, the study partnered with the Ministries of Health, MCHIP program offices, and other stakeholders. The overall objective of the study was to guide quality improvement activities for facility-based maternal and newborn care by determining the frequency and quality of key interventions through direct observation of care. Quality of care was defined based on globally accepted, evidence-based guidelines for maternal and newborn health from the World Health Organization’s manual, *Managing Complications in Pregnancy and Childbirth* [28]. Patient-centered care is an element of these guidelines including provider-client interactions.

Details of the sampling strategy are summarized in Table 1 and reported elsewhere [29–37]. The study was designed to focus on high delivery volume facilities to ensure observers would be present for several deliveries during their visit to each facility. The Kenya survey was designed to be nationally representative with all facility levels represented. Hospitals and health centers throughout the country were also included in Rwanda. MCHIP was conducting (or preparing to conduct) activities to improve maternal and newborn health in all five countries at the time of the survey. In Tanzania, the survey was conducted as a baseline in facilities prior to the start of program activities. The survey in Tanzania was implemented and analyzed separately for the mainland and Zanzibar since they each have their own health systems.

**Table 1:** Summary of samples by country

Country	Facility selection criteria	Number and type of facility	Geographic coverage
Ethiopia	High delivery caseload (≥5)	19 facilities; all hospitals	5 of 9 regions plus Addis Ababa and Dire Dawa
Kenya	Nationally representative by facility type, region, and managing authority	170 facilities; 142 hospitals, 28 health centers/dispensaries	All
Zanzibar	High delivery caseload (≥1) program facilities	9 facilities; 5 hospitals, 4 health centers	All
Rwanda	Hospitals and randomly selected health centers by region	72 facilities; 42 hospitals, 30 health centers	All
Madagascar	High delivery caseload (≥2) and 3 program facilities	36 facilities; 27 hospitals, 9 health centers	17 of 22 regions
Tanzania mainland	High delivery caseload (≥1) program facilities	52 facilities; 12 hospitals, 40 health centers/dispensaries	12 of 25 mainland regions

### Data collection

This paper presents data from the facility inventory survey tool and the labor and delivery observation checklist. The inventory included a complete review of facility infrastructure, presence of necessary equipment and medicines for routine and complicated deliveries, and services offered. Relevant to respectful care, the infrastructure section included a visit by data collectors to the delivery room(s) to determine the level of privacy afforded women. The labor and delivery checklist was a comprehensive tool to capture whether the provider correctly performed key evidenced-based interventions and was divided into four sections: initial client assessment, observation of labor, delivery, and postpartum. The checklist focused on clinical skills such as active management of the third stage of labor, essential newborn care practices, partograph use, and screening for complications.

Ten items concerning provider-client interactions were included in the observation tool; all described actions the provider might take. The five provider actions in initial client assessment were whether the provider greeted the client in a respectful manner, encouraged her to have a support person present, explained procedures before proceeding, informed client of findings, and asked if she had any questions. During observation of labor, the items were whether the provider explained what would happen during labor to the client, encouraged the client to consume food or fluids, encouraged or assisted the client to ambulate and assume different positions, supported the client in a friendly way, and draped the client.

At the end of a case, observers could enter open-ended comments about the quality of care they observed. During training, observers were instructed to use this space to record anything they felt was important in understanding or adding depth to the case, but was not covered in the checklists. If they observed practices that were not to standard, these would be noted in the comments section. No instructions specific to RMC or D&A were given to observers.

Clinical observer training, the survey tools, and study procedures were standardized across countries, with practicing nurses, midwives, and doctors serving as observers. Teams typically spent 2–3 days at each facility working two 8-h shifts per day. Observers followed all consenting clients in the maternity areas during their shift, unless there were too many concurrent clients or a complicated case was prioritized. Paper data collection forms were used in the first survey in Kenya; in following surveys, data were collected using basic smartphones with custom-designed software and built-in data checks. Efforts were made to minimize the effect of observation on provider behavior, i.e. the Hawthorne effect [38], by assuring providers that data collection was anonymous and individual performance would not be reported to their supervisors or shared publically (published reports only refer to aggregate data). Providers were not aware of what topics and items were on the checklists, so they could not prepare in any way. Observers did not visit facilities where they currently or previously worked as clinicians, to minimize the effect of personal and professional relationships.

### Analysis

#### Observational checklist and facility inventory

The unit of analysis was an observation which represents a unique woman, but not a unique provider since providers usually cared for multiple women during the observation period. Data from the facility inventory was linked to individual observations at a given facility in order to present data on privacy by observation (as opposed to by facility). Frequency of occurrence of checklist items and privacy conditions, expressed as a percentage of observations, was calculated by country and for the entire study population. The highest and lowest country percentage for an item is presented as the range. Missing and “don’t know” answers were excluded from calculations. Observers were trained to record a “don’t know” response only in rare occurrences (for instance if they were away from the client during that time or they had trouble seeing what the provider was doing). The overall study was designed to provide descriptive data for multiple countries; differences in sampling strategy resulted in varying coverage of facilities within each country and cross-country statistical tests were not conducted (Table 1). Weighting was applied to data from the Kenya study where the study was designed to be nationally representative. Analysis was conducted using Stata 11 (StataCorp. 2009. *Stata Statistical Software: Release 11*. College Station, TX: StataCorp LP.).

#### Open-ended comments

Not all observations of labor and delivery care included open-ended comments. Those with comments were analyzed with *a priori* codes based on the seven articles of the Charter and the descriptions of these rights and their violations in an advocacy guide for the Charter [18, 19]. Comments in French from Rwanda and Madagascar were translated into English for analysis. Based on the small number of events by category in each country, only aggregate data are presented here. Some observation comments mentioned multiple events, either of the same category or different categories. Number of unique observations with incidents in each of the categories and number of total incidents (differs only where multiple incidents in an observation) are reported. Comments that were particularly striking or summarized commonalities were selected as examples. Comments from the Kenya study were not available for analysis because the paper forms were misplaced.

### Ethical approval

The Johns Hopkins Bloomberg School of Public Health Institution Review Board (IRB) reviewed the study and approved all protocols and consent forms. On a country basis, the study received approval from the Ethiopian Public Health Association IRB, Kenya Medical Research Institute Ethical Review Board, Ministry of Health Ethical Committee in Madagascar, Rwanda National Ethics Committee, Ethical Review Board of the Tanzania National Institute for Medical Research, and Zanzibar Medical Research and Ethics Committee. Informed consent was obtained from the facility director and all participating health providers prior to observation and all clients (or next of kin if necessary) prior to their participation in the study. All providers and clients were assigned id codes to protect their privacy.

## Results

### Characteristics of observations

The facility, provider, and client characteristics of the 2164 labor and delivery observations were very similar across countries (Table 2). Observations were conducted primarily at hospitals in all countries (80 % of deliveries or greater were at hospitals) except in the Tanzania mainland survey, which had a more even mix of facilities with health centers and dispensaries. Ethiopia’s observations were in hospitals. The majority of observed births were conducted by nurses and midwives (87 %) who were female (87 %). In Ethiopia, doctors assisted 20 % of clients and 19 % were doctors in Madagascar. Medical and nursing students and unskilled assistants delivered services in 5 % of observations.

**Table 2:** Distribution of labor and delivery observations by facility, provider, and client characteristics

Observation characteristics	Ethiopia (*N* = 192)	Kenya (*N* = 626)	Zanzibar (*N* = 217)	Rwanda (*N* = 293)	Madagascar (*N* = 347)	Tanzania (*N* = 489)	Total (*N* = 2164)
Health facility type							
Hospital	100.0 %	85.8 %	85.3 %	82.3 %	81.0 %	39.9 %	75.4 %
Health center/ dispensary	0.0 %	14.2 %	14.7 %	17.7 %	19.0 %	60.1 %	24.6 %
Provider cadre^1^							
Doctor	20.3 %	1.1 %	0.5 %	2.0 %	18.7 %	2.5 %	6.0 %
Nurse/ midwife	71.4 %	97.3 %	94.0 %	88.7 %	74.4 %	86.5 %	87.4 %
Student	4.7 %	0.0 %	0.5 %	4.4 %	6.1 %	2.0 %	2.5 %
Unskilled	0.0 %	1.6 %	1.8 %	0.7 %	0.3 %	8.4 %	2.7 %
Other/ missing	3.6 %	0.0 %	3.2 %	4.1 %	0.6 %	0.6 %	1.4 %
Provider gender^2^							
Male	44.3 %	16.7 %	0.5 %	10.9 %	12.1 %	5.0 %	13.5 %
Female	55.7 %	83.3 %	99.5 %	89.1 %	87.9 %	95.0 %	86.5 %
Client gravidity^3^							
Primigravida			23.0 %	37.5 %	31.1 %	22.3 %	28.0 %
Multigravida			77.0 %	62.5 %	68.9 %	77.7 %	72.0 %

^1^ Physician/resident includes: general practitioners, obstetricians, gynecologists, other specialists, residents; assistant medical officers in Tanzania and Zanzibar. Nurse/midwife includes: bachelor of science and diploma nurses, registered and enrolled nurses, bachelor of science and diploma midwives, registered and enrolled midwives, nurse/midwives; nursing officers and MCHA in Tanzania and Zanzibar; paramedics in Madagascar; health officers in Ethiopia. Student includes: medical and nursing students. Non-qualified staff includes: medical attendants, health assistants, and traditional birth attendants. Other/missing category in Kenya includes students

^2^ Gender missing for 43 observations.

^3^ Gravidity not collected in Ethiopia and Kenya, missing for 4 observations

### Right to information, informed consent and refusal, and respect for her choices and preferences (Article 2)

The woman’s right to information was assessed in four checklist items. At their initial consultation (usually admission in labor), providers explained procedures to the clients prior to actions in 62 % of cases (range 38–77 %) (Table 3). Also during the initial examination, it was noted that providers shared their findings with clients in 67 % of observations (range 41–76 %). Scores were similar by country for the two questions, with Kenya and Tanzania mainland having the highest percentages for both actions; clients in Ethiopia received this type of information from providers least often. Only in a third of observations, providers encouraged their clients to ask any questions (range 16–42 %) during this initial interaction. In the first stage of labor in 56 % of observations, the provider explained to the woman what to expect during labor (range 38–62 %).

**Table 3:** Percent of observed clients with respectful maternity care practices

Provider actions during initial assessment	Ethiopia (*N* = 110)	Kenya (*N* = 442)	Zanzibar (*N* = 116)	Rwanda (*N* = 193)	Madagascar (*N* = 277)	Tanzania (*N* = 320)	Total (*N* = 1458)
Greets client in a respectful manner	59.8 %	78.2 %	88.3 %	76.0 %	88.8 %	94.6 %	82.9 %
Don’t know or missing	3	1	13	1	0	7	25
Encourages client to have support person	33.6 %	38.4 %	22.1 %	42.6 %	66.5 %	39.5 %	43.1 %
Don’t know or missing	3	4	12	3	2	9	33
Explains procedures before proceeding	37.7 %	77.0 %	65.0 %	40.4 %	49.1 %	72.1 %	61.9 %
Don’t know or missing	4	2	16	5	4	12	43
Informs client of findings	40.6 %	76.2 %	66.0 %	56.4 %	67.8 %	69.0 %	67.0 %
Don’t know or missing	4	0	16	5	4	10	39
Asks client if she has any questions	16.0 %	35.6 %	21.4 %	42.3 %	28.8 %	26.8 %	30.8 %
Don’t know or missing	4	7	13	4	3	10	41
Provider actions during labor	Ethiopia (*N* = 139)	Kenya (*N* = 571)	Zanzibar (*N* = 120)	Rwanda (*N* = 244)	Madagascar (*N* = 265)	Tanzania (*N* = 306)	Total (*N* = 1645)
Provider explains what will happen during labor to client	37.9 %	61.9 %	44.8 %	58.4 %	53.8 %	60.0 %	56.4 %
Don’t know or missing	7	31	4	11	3	16	72
Provider encourages client to consume food and fluids during labor	40.6 %	61.7 %	62.9 %	47.6 %	35.4 %	79.5 %	56.8 %
Don’t know or missing	6	49	4	11	2	14	86
Provider encourages or assists client to ambulate and assume different labor positions	28.4 %	70.9 %	71.6 %	69.2 %	54.4 %	54.8 %	61.3 %
Don’t know or missing	5	48	4	10	2	16	85
Provider supports client in friendly way during labor	66.2 %	87.1 %	90.5 %	91.6 %	79.5 %	93.2 %	86.1 %
Don’t know or missing	3	29	4	7	2	14	59
Provider drapes client before delivery	44.9 %	24.2 %	47.4 %	68.4 %	85.9 %	46.1 %	48.5 %
Don’t know or missing	3	25	6	7	2	22	65

Three checklist items assessed whether providers promoted the woman’s right to choose evidence-based, respectful, client-focused care practices. Women were encouraged to have a friend or relative with them for support in only 22 to 43 % of cases for all surveys, except for Madagascar with a high of 67 %. More than half of women were assisted or encouraged to ambulate or assume alternative labor positions, except in Ethiopia. Encouragement to consume food and fluids differed greatly among surveys from 35 % in Ethiopia to 80 % in Tanzania.

### Right to privacy and confidentiality (Article 3)

**Fig. 1:**
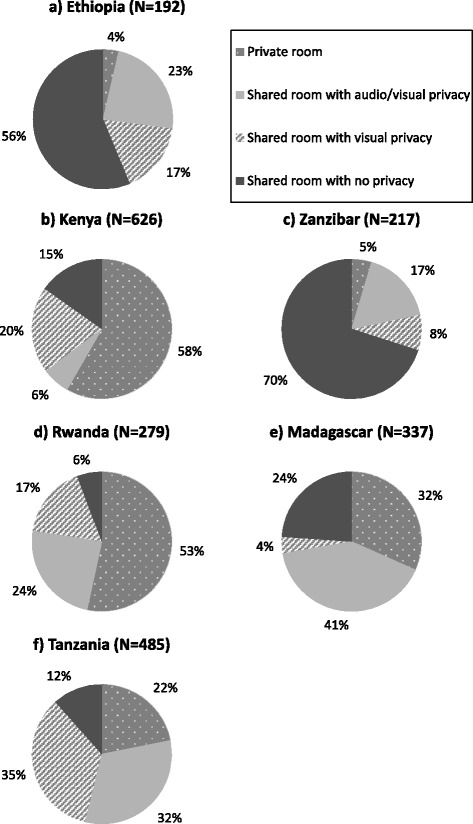
Distribution of observed births according to elements of privacy (*N* = 2164 observations). *Excludes 67 observations missing data

Providers’ use of drapes to preserve women’s right to privacy was varied across surveys. Half or more of clients were draped in Rwanda and Madagascar while in other countries this was less common (24–47 %). In surveys from Tanzania, Kenya, Madagascar, and Rwanda, more than half of women delivered in rooms with auditory and visual privacy (54, 65, 72, and 77 % respectively). In Zanzibar and Ethiopia surveys, most women were in shared delivery rooms with no curtains to separate patients and no way to talk without being overheard (Fig. 1).

### Right to be treated with dignity and respect (Article 4)

Two checklist items assessed provider’s treatment of women with dignity and respect. When first meeting the client, women were offered a respectful greeting by their provider in 83 % of observations (range 60–95 %). Women were supported in a friendly way by their provider during the first stage of labor in 86 % of cases. All countries except Ethiopia scored 80 % or higher on the item for friendly support.

### Characteristics of open-ended comments

Clinical observer open-ended comments were available for analysis from Ethiopia, Madagascar, Rwanda, Tanzania mainland, and Zanzibar. These optional comments were added to 65 % (*n* = 996/1538) of observations. After excluding comments that were indecipherable or related only to survey technology (*n* = 30), 966 observations with comments were available for analysis. Based on the Respectful Maternity Care Charter, 133 observations (14 % of those with comments) described events which were likely violations of women’s rights. Some cases included comments on multiple incidents relevant to an article of the Charter or to multiple articles. A total of 151 events were identified from the 133 observations: there were 18 observations with two items. Table 4 shows the number of events and observations by Charter article.

**Table 4:** Summary of violations of the Respectful Maternity Care Charter as reported in observer comments, by article of the Charter

Respectful maternity care rights	Observations with a violation	Number of violations
Article 1. Right to be free from harm and ill treatment	18	21
Article 2. Right to information, informed consent and refusal, and respect for her choices and preferences	18	18
Article 3. Right to privacy and confidentiality	8	8
Article 4. Right to be treated with dignity and respect	7	7
Article 5. Right to equality, freedom from discrimination, and equitable care	9	9
Article 6. Right to healthcare and to the highest attainable level of health	83	88
Article 7. Right to liberty, autonomy, self-determination, and freedom from coercion	0	0
Total all rights	133^a^	151

^a^ Total does not equal sum of number of observations for individual rights because some observations had multiple violations

### Right to be free from harm and ill treatment (Article 1)

Observers noted harmful treatment in 18 cases (3 with multiple aspects). These included two incidents of slapping or hitting the client (usually in connection to the client not complying with provider orders), for example from an observer in Tanzania: “patient came in second stage of labour pushing now and then, delivered, placenta had difficulties to remove as the mother was not torelant [*sic*] nurss [*sic*] slapped the woman.” Multiple comments described use of fundal pressure, routine episiotomy, and the lack of anesthesia for episiotomies or suturing of tears. For example, an observer in Ethiopia recorded that providers at the facility “used episiotomies for all primi gravida mothers.”

### Right to information, informed consent and refusal, and respect for her choices and preferences (Article 2)

Comments on 18 observations related to this right including six times when providers failed to provide information. Within this category, other examples are when women were restricted in their choice of birth position and movement (*n* = 5) and not allowed fluids during labor (*n* = 2). This incident described by an observer in Rwanda (translated from French) demonstrates how a situation escalated to include other violations: Each time she had a contraction and wanted to give birth in a squatting position, two doctors intervened in vain to convince her to labor in the conventional position. They pressured her, even hit her so that she would accept to climb in the bed. In a case in Ethiopia, an observer reported that “no one provided components of mother frindly [*sic*] care, nothing had been informed regarding progress & finding to the client.”

### Right to privacy and confidentiality (Article 3)

Eight comments were all related to lack of physical privacy during labor and delivery including a woman in Zanzibar “laying naked on the floor” and cases where there were no sheets or drapes for the mother.

### Right to be treated with dignity and respect (Article 4)

Seven comments related to this right noted unfriendly, disrespectful attitudes. During a case in Rwanda where the woman required surgery which was delayed waiting for appropriate staff and supplies, the observer noted the anesthetist yelling at the woman in labor (translated). Soiled linens were being re-used including where the provider was “[c]leaning the vagina with durty [*sic*] client clothes” (observer in Tanzania).

### Right to equality, freedom from discrimination, and equitable care (Article 5)

Observers noted eight cases where client’s access to necessary medications was affected by lack of finances. This resulted in denial and/or delays in receiving uterotonic for prevention of postpartum hemorrhage or augmentation of labor. From the comments, it is not clear in most cases whether the family was requested to pay for medications based on facility or national policy, lack of supplies, or as informal payments. In a ninth incident there was a woman in need of referral for complicated delivery who was not sent because of cost; luckily she and her baby were successfully treated at the facility (Madagascar).

### Right to healthcare and to the highest attainable level of health (Article 6)

The most frequent violated right in open-ended comments was the right to care in 83 observations (five with multiple incidents). Of these 83 cases with abandonment or delays in care, a primary issue was clients who were monitored infrequently or not at all during labor and postpartum (28 cases). In eight cases, comments indicate that there were not enough providers or that a single provider was caring for multiple patients. Four women delivered without a provider and in two of these cases, the only provider was busy with another patient: “This woman delivered on her own. The midwife was attending another client” (observer in Zanzibar). There were many delays in decision-making reported - whether to perform a caesarean-section (CS) or assisted delivery, or whether to call another provider in for a consultation - as well as delays in taking action, for instance waiting while other clients are attended, or for other providers to arrive. Comments related to some cases where the newborn did not survive suggest that neglect and delays in care were a contributing factor: “patient transferred from…health centre with prolonged labour and fetal distress…taken for CS after 3 h 15mins. Baby noted to be fresh [stillbirth]…delays observed including decision to do elective CS before this case” (observer in Tanzania). Seven observations noted delays in starting resuscitation for an asphyxiated newborn; sometimes supplies were at another location, a specialist was needed, or the provider was delayed in identifying the need for resuscitation.

## Discussion

This paper describes health provider care practices using the seven universal rights of childbearing women defined in the White Ribbon Alliance’s Respectful Maternity Care Charter. This analysis is one of the first with a focus on measuring respectful care through direct observation of labor and delivery. Over two thousand observations were conducted in five countries using structured, standardized observation checklists based on World Health Organization guidelines. Due to the size and scope of the study, these results provide a broad overview of provider-client interactions in diverse settings in Sub-Saharan Africa. Encouragingly, women overall were treated with dignity and in a supportive manner by providers, but specific issues were identified that need to be addressed at the health systems level, including inadequate interpersonal communication by providers, abandonment and delays in care including a lack of routine monitoring, inadequate privacy protection, and in some cases, physical and verbal abuse.

Results from the observation checklist indicate that provider communication and information sharing skills were lacking during the study and prevented women from fully realizing their right to information, informed consent and refusal, and respect for their choices and preferences. Many women did not have procedures or the labor process explained to them and did not hear about the findings of exams. The least observed checklist item was whether the client was asked if she had any questions, with a prevalence of 16 % in Ethiopia and high of only 42 % in Rwanda. A provider who asks for questions (and listens to and answers them) is providing an important opening for the client to establish herself as an informed and active participant in the care process. In a study of D&A in Ethiopia, women also reported a similar lack of client-provider information sharing: 63 % of women were not encouraged to ask questions, 43 % did not have procedures and the labor process explained, and 32 % received no update on the progress of their labor [25].

As providers transition from a disease-oriented approach to a patient-centered one, they may need to build new interpersonal skills or improve existing ones. Educational interventions are an effective method of changing how providers communicate [39]. A Cochrane systematic review of training programs aimed at providers to improve patient-centered approach reported a positive effect on provider consultation skills [40]. However, no middle or low income countries were included, the providers were primarily specialists or context was a specific disease, and reported outcomes were heterogeneous (shared decision making, empathy, length of interview, etc.). Further research is needed understand whether these interventions are effective for improving interpersonal skills of maternal care providers in this context.

Observers’ open-ended comments were a rich source of details, providing evidence of poor behaviors that were not explicitly asked in the checklist. Delays in care and abandonment of women during labor, delivery, and postpartum were the most frequent type of respectful maternity care rights violation noted in the comments (over 60 % of cases that classified as violations). Reports of women feeling ignored and neglected during facility delivery are common in the literature [22]. Although definitions were variable, the four studies identified earlier as providing estimated prevalence of D&A from Ethiopia, Nigeria, Kenya, and Tanzania reported neglect and abandonment in 9–29 % of women [24–27]. Especially concerning in the present study were comments describing situations with the potential to become life-threatening for mother and newborn. These include reported delays in referral or performing cesarean sections or newborn resuscitation and women delivering without the help of a provider. Nine percent of women in the Nigeria study and 4–5 % of women in Tanzania reported delivering alone [26, 27].

Observer comments identified lack of resources, including staff shortages, as key reasons for abandonment and neglect. These five countries face severe staff shortages with the density of skilled health workers (midwives, nurses, and physicians) per 10,000 population far below the WHO threshold of 22.8 [41]. Basic infrastructure is also lacking; nationally representative surveys in Ethiopia, Kenya, Rwanda, and Tanzania reported no electricity available in 14, 26, 18, and 50 % of facilities, respectively [42]. The current study found that availability of essential supplies for deliveries at visited facilities was lacking (range 20–57 % by country for presence of sterile scissors or blade, disposable cord ties or clamps, suction apparatus for use with catheter, and skin antiseptic) [30–35]. The important role of lack of resources as well as absence of accountability policies and facility culture in mistreatment of women at facilities was identified in a recent mixed methods systematic review [22].

Long term exposure of providers to intractable health system problems can lead to poor morale, compassion fatigue, and disrespectful treatment of clients and fellow providers [43–47]. There is a need to systematically examine how these constraints commonly found in low income countries foment D&A and act as a barrier to respectful care. This research should inform efforts to reorganize care and put in place plans to encourage respectful care at the health systems level. For example, support for respectful care could be achieved by improvements in facility infrastructure for privacy and to provide dedicated space in the delivery room for birth companions. Since this is a developing area, few relevant interventions have been developed or tested. Some of the strategies suggested for interventions include greater health systems accountability, policy and regulatory approaches, training and supportive supervision, ethical codes of conduct, and community-level awareness programs for women [45, 48, 49]. Standards-Based Management and Recognition (SBM-R), which uses detailed performance standards to assess health facilities as part of a change management strategy for improvement, has demonstrated positive impacts on maternal newborn care quality and also may be a useful approach for respectful care [50].

A particular concern for those conducting research on RMC and D&A is how to determine which events or situations qualify as respectful or abusive. An outsider seeing women giving birth two to a bed may find this situation unacceptable, but local providers and clients may view this as part of the typical experience. Our approach in the present analysis was to use the standards in the Respectful Maternity Care Charter because the overall Quality of Care study was based on international standards. Freedman et al. proposed a research definition of D&A to include interactions and facility conditions that local consensus considers D&A or that women experience as D&A [51]. As awareness and norms change over time, they expect the definition to expand to include human rights standards. These two approaches can yield different results since some items identified here as negative behaviors by international norms may not have been seen as disrespectful in the local context, by women experiencing them, or by their providers.

### Limitations

A limitation of the study is that the data collection tool was not designed specifically to examine RMC. There were no checklist items related to respectful care during the second and third stage of labor or postpartum and certain concepts such as consent for procedures and detention of mothers were not covered at all. Regarding the open-ended comments, the results should be interpreted carefully since observers were not specifically trained or sensitized to the concept of respectful care and the decision whether to enter a comment for a given observation was at their own discretion. Since our observers were health providers, the comments were also likely influenced by their professional training and experiences. Future research should consider incorporating comments as a fixed element with appropriate training on standards. Revised checklists with specific questions on delays/abandonment and other issues suggested from the analysis would also be useful.

The overall study was designed to provide descriptive data and collected limited data on characteristics of facility, provider, and client. Concurrent activities to improve maternal and newborn health were likely taking place in the survey countries before and during the survey and these may have impacted results. In addition, the facility sample in each country varied considerably in terms of regional coverage and level and size of facilities and thus should not necessarily be considered generalizable to the entire country. Differences between countries may reflect the sampling strategy, or other unmeasured factors rather than true differences. Where possible, future research should utilize a sampling strategy that better represents coverage of facilities of certain types and facilitates comparisons. Research that explores associations between facility, provider and client-level factors and the observed elements of respectful maternity care, or lack of it, would be valuable. Lastly, we cannot ignore the possible impact of observation on provider behavior (Hawthorne effect), although efforts were made to minimize its impact. This may have caused an underestimate the true extent of the issues explored here.

## Conclusions

Efforts to increase use of facility-based maternity care in low income countries are unlikely to achieve the desired gains if there is no improvement in quality of care provided, especially elements of respectful care. This analysis identified insufficient communication and information sharing by providers as well as delays in care and abandonment of laboring women as deficiencies in respectful care. Failure to adopt a patient-centered approach and a lack of health system resources are contributing structural factors. Further research is needed to understand these barriers and develop effective interventions to promote respectful care in this context.
